# Ontology-Based AI Design Patterns and Constraints in Cancer Registry Data Validation

**DOI:** 10.3390/cancers15245812

**Published:** 2023-12-12

**Authors:** Nicholas Nicholson, Francesco Giusti, Carmen Martos

**Affiliations:** 1 European Commission, Joint Research Centre (JRC), 21027 Ispra, Italy; 2 Belgian Cancer Registry, 1210 Brussels, Belgium; francesco.giusti@registreducancer.org; 3 Rare Diseases Research Unit, Foundation for the Promotion of Health and Biomedical Research in the Valencian Region (FISABIO), 46020 Valencia, Spain; carmen.martos@fisabio.es

**Keywords:** data validation, knowledge representation, ontology-based AI, ontology design patterns, machine reasoning, cancer registries

## Abstract

**Simple Summary:**

Ontology-based AI promises a more flexible and cost-effective approach to the validation of cancer registry data over traditional approaches and may allow a more straightforward means of federating centralised data-harmonisation processes. The advantage of using ontologies is that they unite the conceptual and logical aspects of a given domain and, therefore, provide an inherent means for expressing data validation rule sets. The idiosyncrasies of data validation, however, impose certain constraints on the ontology structure of AI-based reasoning and require design patterns that are not immediately obvious. The design patterns presented in this work are generic to other domains beyond cancer registries and serve to point out the types of issues that validation ontologies have to confront, with proposed solutions for tackling them.

**Abstract:**

Data validation in cancer registration is a critical operation but is resource-intensive and has traditionally depended on proprietary software. Ontology-based AI is a novel approach utilising machine reasoning based on axioms formally described in description logic. This is a different approach from deep learning AI techniques but not exclusive of them. The advantage of the ontology approach lies in its ability to address a number of challenges concurrently. The disadvantages relate to computational costs, which increase with language expressivity and the size of data sets, and class containment restrictions imposed by description logics. Both these aspects would benefit from the availability of design patterns, which is the motivation behind this study. We modelled the European cancer registry data validation rules in description logic using a number of design patterns and showed the viability of the approach. Reasoning speeds are a limiting factor for large cancer registry data sets comprising many hundreds of thousands of records, but these can be offset to a certain extent by developing the ontology in a modular way. Data validation is also a highly parallelisable process. Important potential future work in this domain would be to identify and optimise reusable design patterns, paying particular attention to avoiding any unintended reasoning efficiency hotspots.

## 1. Introduction

Population-based cancer registries collect data on cancers occurring in a defined population and are an essential element in cancer surveillance [1]. They are also a useful reference for cancer epidemiology. Data validation is an important process in the work of registries since statistical bias can lead to erroneous conclusions with potentially far-reaching implications. Apart from the more simple and generic types of validation checks, tailored data validation software is, however, often necessary and leads to costly and complex development and maintenance processes [2]. At a generic level, data validation requirements are similar and revolve around tasks that need: (a) to describe the validation rules in unambiguous terms; (b) to develop the associated validation software and test-data sets; (c) to manage the change control and maintenance processes; (d) to handle and coordinate the various version releases; and (e) to ensure the correct application of the data validation procedures.

### 1.1. Centralised Validation Process of Cancer Registry Data

These validation requirements underlie the centralised validation process of data collected from cancer registry members of the European Network of Cancer Registries (ENCR) for the purpose of providing accurate and comparable cancer indicators across Europe [3]. In this process, data are submitted to the central processing point from the individual European cancer registries. Owing to the fact that health services are regionally based in a number of EU Member States, there are over 150 cancer registries. The data are submitted in a common data template prescribing the data variables and their permissible ranges and formats. The data are thereafter checked against a predefined and agreed set of data rules using dedicated software [4] that outputs any errors found to a data log. The data logs are then returned to the associated cancer registries for correction/verification. Once this process is completed, the data are aggregated for public release on the European Cancer Information System (ECIS) data browser [3].

The centralised process has a number of drawbacks. One major issue is that it incurs time delays in the availability of data and compromises their usefulness in feeding back into health-policy initiatives. The delays are inherent in the process of coordinating data collection and validation across so many different entities and are compounded by administrative and bureaucratic processes relating to increasingly stricter EU data protection laws that are often implemented differently according to national/regional policies. Another issue relates to the centralised collection point itself. The ENCR is not a legal entity in its own right and receives no funding for its operation. The European Commission’s Joint Research Centre currently provides a type of secretariat to handle the data-cleaning operation but relies on the knowledge and experience of staff who are often recruited on temporary contracts, resulting in potential risks of continuity. The difficulties and time delays associated with the ENCR centralised collection and validation of sensitive data could be alleviated by devolving these activities to the local cancer registry level. A process federation of this type, however, would require a straightforward and manageable mechanism for handling the individual steps of the data validation lifecycle. Ontologies provide a possible means of accomplishing this.

### 1.2. Role of Ontologies

The word ontology derives from two Greek words and, crudely interpreted, means the study of things that exist. In relation to this definition, computer ontologies express a knowledge domain via a set of representational primitives (such as classes, attributes, and relationships) [5]. Classes represent specific entities in the knowledge domain and are related to each other via a set of well-described relationships. For example, in the cancer registry domain, classes might represent such concepts as topography, morphology, grade, stage, etc. Relationships might then express the dependencies between such classes; for example, specifying which morphologies could be associated with which topographies, and which grades can be used with which morphologies, etc.

In contrast to databases, ontologies are able to integrate the conceptual aspect of the domain (describing the semantics of the domain and the scope of the model) and the logical aspect (describing the structure of the information) [6]. A particularly useful aspect is that the associated expressions can be represented in description logics (DLs) and, therefore, benefit from a formal representation as well as the predisposition to AI-based reasoning, giving rise to the term ontology-based AI.

The ENCR data validation rules [7] describe tumours according to the international classification of diseases for oncology (ICD-O-3) [8] and the internationally recognised TNM classification for solid tumours (where “T” encodes the size and tissue invasion of the tumour; “N”, the number of local lymph nodes involved; and “M” any distant metastases) [9]. ICD-O-3 describes the tumour in terms of: topography (associated with 330 codes specifying the site of the tumour); morphology (associated with just under 2000 codes specifying the type of tumour); and behaviour (associated with six codes describing the nature of the tumour). The TNM classification identifies the stage of the tumour; a further ICD-O-3 term describes the grade (associated with eight codes specifying the extent of the tumour); and the basis of diagnosis is an additional code set comprising eight codes describing how the tumour was diagnosed. The ENCR validation rules stipulate the permissible combination of the ICD-O-3 codes and their restrictions on other factors such as age, sex, and basis of diagnosis. While some ENCR rules apply internationally accepted rules (such as the TNM classification and the definition of multiple primary tumours), others are agreed within working groups comprising members with specific expertise from the European cancer registries. The outputs from the working groups result in a series of recommendations for adherence by the European cancer registry community. The ENCR rules form the basis of the data-cleaning checks, and divergences from the rules trigger error and warning messages in the resulting data logs that are sent back to the registries for verification.

### 1.3. Tabular Presentation of the ENCR Validation Rules

The ENCR data validation rules are generally presented in tabular form [7]. For reasons of clarity, a tabular representation is only able to show a limited number of dependencies at a time, and the whole set of dependencies therefore have to be displayed across a number of tables. As an example, morphology is related to practically all other variables (topography, behaviour, grade, stage, age, and basis of diagnosis). It becomes a laborious task to check each of these dependencies separately from each table and constructing a test-case data set for verifying the rule-checking software is particularly tedious. Any valid combination of variables can only be verified after cross-checking each of the tables in turn. An ontological representation of the rules has the advantage that all the dependencies are visible at the same time for any given class; it is, moreover, applicable to any data domain where dependencies between variables can be captured by a set of rules.

Initial work showed the viability of applying ontology-based AI to cancer registries’ data validation requirements [10] but also highlighted the challenge of achieving good ontology designs that are serviceable to the needs of federating a relatively complex process. We present the design principles taken towards the realisation of this aim that may hopefully serve as a potential reference to other ontology developers facing similar issues. In the newly evolving field of ontology engineering, there is a sparsity of guidelines apart from some general recommendations [11,12,13,14]. Moreover, the information needs of an ontology may require justifiable compromises in the application of such recommendations. Whereas design patterns are becoming increasingly available [15], the needs of data validation applications tend to be quite specific.

## 2. Methodology

We used the web ontology language (OWL) and the Protégé ontology editor for designing the ontologies to validate the cancer registry data. OWL forms part of the semantic web architectural stack and can operate relatively seamlessly with other components in the stack, which is an advantage for semantic operability. With Protégé, it is a straightforward task to create classes and the relationships between them. The expression editor uses Manchester OWL syntax [16], which is more intuitive and easier to understand than DL-style syntax.

By creating class hierarchy trees, it becomes a manageable task to assign relationships for the 2000 plus morphology codes. A relationship can be assigned at a relevant super-class level that is inherited by all the corresponding subclasses. For example, by creating two separate morphology code trees associated with haematological-type morphologies and solid-tumour-type morphologies, one can treat each block of codes as a whole and still distinguish between any of the individual subclassed codes in the two trees.

Using Protégé, it is possible to choose from a selection of DL reasoners for verifying the logic of the axioms throughout the development process. Prior to running the reasoner, the ontology consists only of asserted axioms (including classes, class trees, and expressions); after running the reasoner, the ontology consists of the original asserted axioms as well as the results arising from logical inferences drawn from those axioms. The logical inferences made by the reasoner include further class hierarchies that are not defined a priori in the asserted ontology. The process of determining other classification trees in the reasoning process is called class subsumption (a class is subsumed by another class when it is inferred to be a subclass of that class). This automatic class subsumption is the means of determining whether a cancer case record or part of it is valid or not. However, in order to ensure class subsumption, a number of design constraints may need to be imposed on the ontology for reasons described in the following sub-section.

### 2.1. Expressivity of Description Logics

DLs are a family of languages based on first-order logic that are described by their expressivity (or types of operations they support) [17]. Higher expressivity languages have greater modelling power but also increase computational requirements. An important aspect of DLs concerns decidability (deriving a correct answer from the reasoning process), and most of the expressivities supported by the OWL ensure this. The basic language is AL (associative language), which has a limited set of operators that allow axioms to specify the negation and intersection of classes and to construct certain containment relationships between classes. It comprises the operators: atomic negation “not” (⌐), concept intersection “and” (⊓), universal restrictions “only have relationships” (∀), and limited existential quantification “at-least-one relationships” (∃). The addition of complex concept negation (C), including concept disjunction “or” (U) and full existential qualification (ε), to provide the ALC (attributive language with complements) furnishes a relatively powerful modelling language. The label S is used as a substitution for ALC with transitive roles (allowing inferences of direct relationships between otherwise indirectly connected classes). Higher expressivities include inverse properties (I), role hierarchy (H), nominals (O), cardinality restrictions that allow an exact number of relationships with other entities (N), and qualified cardinality restrictions (Q) that allow cardinality restrictions on specific classes of objects. These additional features provide a richer modelling language. 

Tools are available for machine reasoning on the basis of axioms written in DL. The result of the reasoning process is an expanded classification hierarchy of the ontology based on the class subsumption decisions logically inferred by the reasoner from the asserted axioms. In terms of algorithm complexity, most logic-reasoning processes are hard, and computational performance demands account for one of the main current drawbacks, requiring circumspection in how an ontology is designed. By limiting the expressivity to the εLH (existential language with role hierarchy, i.e., including concept intersection, existential restrictions, and sub-properties), classification of the ontology can be performed in polynomial time (PTIME) [18], which is advantageous for processing performance. The higher expressivity of SHIQ has a worst-case complexity of EXPTIME [19] and SROIQ of N2EXPTIME [20], which, in the worst-case scenarios, would make them unusable. While the introduction of highly optimised implementations of Tableau-based algorithms [21] has enabled the use of higher expressivities in practical applications, care nevertheless has to be exercised to limit the expressivity as far as possible, especially for ontologies involving tens of thousands of axioms. Reasoners have different strengths in relation to different types of ontology structures, but it is not immediately clear which structure is more amenable to a given reasoner [22,23,24,25,26,27,28,29].

Moreover, two particular properties of DL provide challenges for modelling validation rules; one is the open-world assumption and the other is logic monotonicity. The open-world assumption considers that any information not explicitly stated a priori can only be assumed to be unknown. While this view is necessary from the perspective of having to model incomplete knowledge, which is a requirement for knowledge derived from web resources, it has important consequence for how classes are subsumed by others. Data validation is a process better described by the closed-world assumption, where information not explicitly known to be true is considered to be false. Logic monotonicity is a consequence of semantic entailment and, in practical terms, prevents the overriding of default values—if a default value is asserted in DL, it cannot thereafter be modified independently of how much extra information is provided that may warrant its modification. This presents difficulties for scenarios commonly encountered in data validation rules, for example when a default condition is specified at a super-class level and one or more subclasses present exceptions to that condition; it is not possible in these cases to override the default condition with another condition for the exceptional subclasses.

### 2.2. ENCR Ontology Suite

The ENCR data validation process can be handled with a suite of three main ontologies. While these three ontologies may be integrated into one large ontology without difficulty, the reasoning performance would be unduly affected. Moreover, given that not all validation checks are required for all of the tumour records, it makes sense to use only those ontologies that are relevant to a given set of cancer cases.

**Figure 1:**
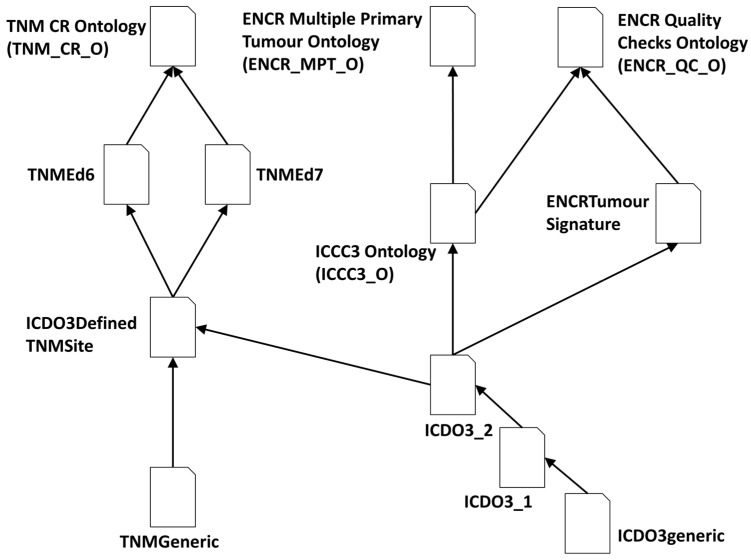
Ontology import tree for the three main ontologies—TNM_CR_O for validating TNM stage, ENCR_MPTO_O for validating multiple primary tumours, and ENCR_QC_O for validating all other rules.

One of these three main ontologies contains the axioms required for validating rules affecting all cancer case records; the other two ontologies are used for validating subsets of cases that either have TNM-stage associated information or are instances of cases with multiple primary tumours. Multiple primary tumours are defined as a diagnosis of two or more independent primary tumours in the case records of a person with cancer. The conditions for the independency of tumours for cancer registration are defined by a set of international rules [30] for the consistent recording and reporting of cancer cases for comparability purposes among population-based cancer registries. All three ontologies draw from a number of common base ontologies (OWL files), thereby facilitating maintenance aspects. Figure 1 shows the ontology import tree of the three ontologies. The TNMEd6 and TNMEd7 imports contain the axioms specific to the TNM classification editions 6 and 7, respectively. The ICDO3DefinedTNMSite import consists of axioms related to the TNM sites expressed in terms of the ICD-O-3 codes, and the ICCC3_O import contains the morphology categories defined in the third edition of the international classification of childhood cancer (ICCC). The ENCRTumourSignature import consists of the axioms defining permissible combinations of morphology and topography codes, and the two generic-named imports consist of common axioms used by the different TNM editions (TNMGeneric) and ICD-O-3 versions (ICDO3generic). Together, the three imports ICDO3generic, ICDO3_1, and ICDO3_2 (ICD-O versions 3.1 and 3.2, respectively), constitute the ICDO3 ontology (ICDO3_O).

### 2.3. Modelling the ENCR Data Validation Rules

With regard to the design constraints of the ontology modules, a critical aspect was how best to specify the associations between classes that had bi-directional interdependencies (e.g., topographies and morphologies) whilst ensuring their consistency across different modules and allowing the necessary class subsumption outcomes, especially in relation to the open-world assumption of DL. The answers to such questions must take also into account the different DL expressivities required by each ontology in the suite.

While most ENCR validation rules can be modelled in the TBox (terminology component of the knowledge base), some may also require an ABox (assertion component of the knowledge base). The TBox of a knowledge base can be considered similar to the schema of a database; it contains the classification trees of the classes and the relationships between them. The Abox, in contrast, contains specific instances of classes defined in the TBox. Working only with the TBox is generally less computationally expensive than working with an Abox as well. 

Rules needing an ABox tend to require a higher expressivity DL and, consequently, may dictate the formulation of the properties used in the wider ontology space. As an example of this, a tumour may be expressed as a permissible combination of a morphology code and a topography code. In the multiple primary tumour ontology [31], for reasons related to the need for qualified cardinality restrictions (explicitly requiring an ABox), an appropriate axiom for a specific tumour defined by the ICD-O-3 morphology code 8082 and topography code C051 would be given by Equation (1):

∃hasMorphology.M_8082 ⊓ ∃hasTopography.C051 ⊑ Tumour(1)

The same axiom written in Manchester OWL syntax takes the more readable form:

(hasMorphology some M_8082) and

(hasTopography some C051) SubClassOf Tumour

This axiom loosely states that a hasMorphology relationship with the morphology code 8082, and a hasTopography relationship with the topography code C051together, constitute a tumour (the intersection of these existential relationships with the codes shown would, in the reasoning process, be subsumed by the tumour class).

#### 2.3.1. Modelling Tumour Signature

The way in which axiom (1) is expressed influences how morphology and topography axioms are formulated in other parts of the ontology suite. Without the constraint of qualified cardinality restrictions, the way of associating the permissible set of topography and morphology codes could possibly be represented by the pattern of Equation (2):

C051 ⊑ M_8082(2)

which simply subclasses the topography code C051 under the morphology code 8082. While this expression makes it particularly easy in an ontology browser to see the whole set of topography and morphology associations from the asserted class hierarchy, it would insinuate that topography is a type of morphology, which is not the case. An alternative expression that breaks the direct class–subclass relationship would be:

C051 ⊑ ∃hasMorphology.M_8082(3)

which states that topography code C051 is a subclass of a hasMorphology relationship with morphology code 8082. This axiom would allow a user to derive both sets of associations, either by looking at the super-classes of the topography to see the corresponding morphologies or by running a DL query on the ∃hasMorphology existential relation of the morphology code to see the associated topography subclasses. There are two problems, however, with this formulation. The first problem is that it would require the topography code in a cancer case to be expressed in two different ways—first, as a simple or primitive class to derive the information from Equation (3) and, second, as an existential relation for the multiple primary tumour ontology in the formulation of Equation (1). The second problem is that it would encounter difficulties when specifying super-classes of morphologies (which is convenient for collectively referring to a large number of morphology subclasses). The morphology code M_8082 is subclassed from the set of morphology codes M_808 (which is itself subclassed from a further more general set). If one were to specify such a general morphology class in Equation (3) and execute a DL query to find the topography codes associated with the specific morphology code M_8082, the reasoner would not find the C051 association due to reasons of the open-world assumption of DL—there would not be enough information to warrant the subsumption of the topography class by the morphology class.

Reformulating Equation (3) as Equation (4) would circumvent the first problem (different formulations for the same concept) but not the second (class subsumption).

∃hasTopography.C051 ⊑ ∃hasMorphology.M_8082 (4)

Equation (4) subclasses a hasTopography relationship with the topography code C051 from a hasMorphology relationship with the morphology code 8082. This axiom would also make it more difficult to visualise the associations between topography classes and morphology classes since both sides of the expression consist of complex classes (classes within expressions). Moreover, neither of the patterns of Equation (3) or Equation (4) is able to model the validity of a combination of a specific topography and morphology without at least defining role compositions and incorporating inverse relationships to show their bilateral dependence. While role chain inclusions remain tractable in εL [32], inverse roles increase language complexity and generally lead to unsatisfactory performance using Tableau-based reasoners [33]. A more serious issue is that using inverse roles together with qualified cardinality restrictions leads to un-decidability [34], whereby the reasoner cannot return an answer. 

A solution is not to define any relationship at all between the two class types but, instead, to decouple them via a duplicated or “surrogate” class hierarchy that mirrors the class hierarchy of one or other of the two class types. In this way, the morphology code classes could be subclassed from the surrogate topography classes according to the pattern of Equation (5):

M_8082 ⊑ C051Morph(5)

where, now, the morphology code is subclassed under the C051Morph class, which represents a surrogate topography class of C051. Using such a pattern still allows the associations in the visual terms of the class hierarchies to be made between the morphology and topography codes (via the similarly named surrogate topography codes). A further advantage is that the axiom in Equation (6) could then be used to expand the existential relationship ∃hasMorphology to the whole set of morphology codes subclassed from C051Morph (of which the morphology code 8082 in Equation (5) is just one example), thereby allowing the general formulation of a set of axioms specifying the valid combinations of topography and morphology codes, following the pattern of Equation (7).

∃hasMorphology.C051Morph(6)

∃hasMorphology.C051Morph ⊓ ∃hasTopography.C051 ⊑ VALID_TumourSignature (7)

**Figure A1:**
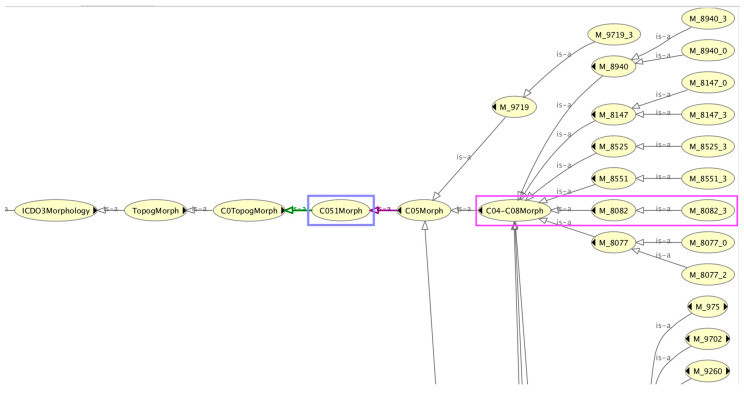
Part of the classification tree of the surrogate topography class C051Morph, showing its subsumption of the morphology class M_8082_3 (highlighted in the boxed sections).

The intersection between a hasMorphology relation with any subclass of C051Morph and a hasTopography relation with any subclass of C051 would result in the subsumption of a valid tumour signature condition. An important point to notice from the first term of Equation (7) is that the surrogate topography codes are in fact morphology codes (unlike the situation in Equation (2)). The surrogate topography classification tree is, then, essentially a set of morphology codes organised following the structure of the topography classification tree (an example is shown in Figure A1 of the Appendix A). This would suggest a generic pattern in these types of problems where one needs first to create a duplicate set of surrogate classes of type B (but named and arranged according to the classification tree of type A) and, thereafter, to subclass the type-B classes under the surrogate code tree.

A drawback to this approach is the entanglement of the asserted ontology hierarchy (via multiple inheritance), which is not considered good practice owing to the fact that the trees cannot be split into disjoint branches to provide a more modular ontology [13]. While entanglement could be removed by subclassing existential relationships rather than primitive classes (c.f. the patterns of Equation (3) and Equation (2), respectively), the ease of visualising the associations—at least in an ontology editor such as Protégé—would then be lost.

#### 2.3.2. Modelling Tumour Behaviour

A similar issue arises from the association of tumour behaviour with morphology. While some validation rules are only applicable to certain tumour behaviours, others are indifferent to them. It is, in any case, useful to know which morphologies are associated with any particular tumour behaviour. Given that a morphology may have more than one behaviour, it would seem appropriate to subclass the behaviours in some manner from the morphologies. Subclassing the behaviour codes directly under the morphology codes would confuse the meaning of tumour behaviour when using an existential relation ∃hasMorphology (at least without defining the associated role composition hasMorphology ∘ hasBehaviour), in much the same way as discussed in the previous section for the relationship between morphology and topography classes. Given the limited number of behaviour codes associated with each morphology code, we opted for creating a set of primitive classes under the morphology codes with the same names as the morphology code but appended with a number corresponding to the behaviour code, as in Equation (8) for behaviour code 3 (malignant behaviour). Although this increases the class structure both in size and depth, there is generally only a maximum of two or three behaviours per morphology code.

M_8082_3 ⊑ M_8082(8)

However, since the reasoner is unable to make inferences on the name of classes, an extra axiom, Equation (9), is needed to associate logically the behaviour code with the morphology:

∃hasMorphology.M_8082_3 ⊑ ∃hasBehaviour.BehaviorCode3(9)

The axiom in Equation (9) subsumes the class described on the left-hand side of the expression under the class of the right-hand side of the expression, which is relevant for axioms requiring knowledge of behaviour. Thus, whenever a hasMorphology relationship is used with a behaviour-appended morphology code, the expression will be subsumed by the reasoner under the associated hasBehaviour axiom.

This type of construct, which defines a complex subclass (left-hand side of Equation (9)), is termed by Protégé as a general concept inclusion (GCI). The approach in this case, however, costs an extra few thousand axioms (at least two axioms for each morphology code). Moreover, it requires axioms in all the other modules to specify the morphology code appended by the behaviour code, where knowledge of the behaviour is necessary (such that the class subsumption can be realised). 

The important point to consider in a scalable ontology is how certain parts of the ontology might constrain the formulation of the axioms used in other parts of the ontology. ABox axioms in particular would form a good starting point from which to consider these constraints due to the greater restriction they place on class usage.

#### 2.3.3. Overcoming Monotonicity Constraints

There are few ways around the limitations imposed by monotonicity on rules that are framed in terms of a default value for the majority of cases and another value for exceptional cases. Creating axioms that only flagged the exceptional cases would create ambiguities in knowing whether a non-flagged general case was valid or invalid. As a design decision, we opted to subclass a global axiom capturing all the conditions of the rule under a default value (for example, default valid or default invalid) and then to subclass the exceptions under a definitive exception flag. This would mean the user/application having to check not only the default condition class but also the presence or absence of the exception class before ascertaining whether the default condition was, in fact, correct or not. Equation (10) shows a global (invalid default value) rule applied to all cancer cases diagnosed from a clinical investigation (basis of diagnosis code 2), through a direct subclass relationship:

∃prevalidatedBoD.BoDcode2 ⊑ InvalidBoDDefaultCase(10)

This would mean that all basis of diagnosis codes of value 2 are, by default, subsumed under an invalid basis of diagnosis condition. In order to flag an exception to this default condition (for example, a basis of diagnosis code 2 is valid for a morphology code 8000 and behaviour code 1 in the case of tumours of the central nervous system), an axiom along the lines of Equation (11) can be used:

∃prevalidatedBoD.BoDcode2 ⊓ ∃hasMorphology.M_8000_1 ⊓ ∃hasTopography.C751 ⊑ VALID_BoD(11)

The axioms modelling the various scenarios described in the preceding text are summarised, for convenience, in Table A1 of the Appendix A. 

## 3. Results

The figures in this section show how the ontology suite is able to validate hypothetical cancer registry cases according to a selection of the validation rules. The yellow highlighted lines in the class description windows at the top of each figure relate to the inferences made by the reasoner. The non-highlighted lines relate to the information passed to the reasoner. The lines highlighted in green in the windows beneath the class description windows depict the traces provided by the reasoner for reaching the associated inference. The inferences illustrate some of the patterns referred to in Section 2.

### 3.1. Validation Results Using the General ENCR Quality Check Ontology (ENCR_QC_O)

**Figure 2:**
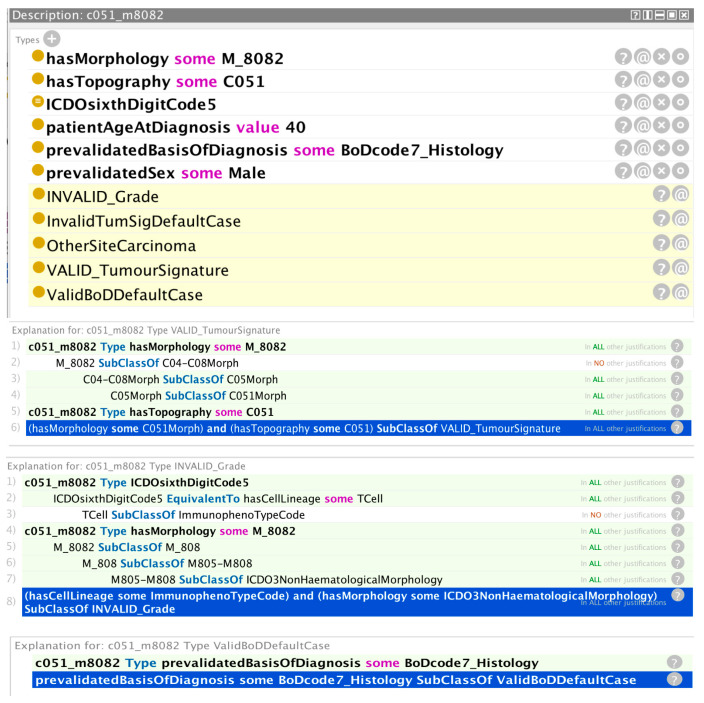
Inferences made by the reasoner (highlighted yellow text) on the basis of the asserted information provided (non-highlighted text) and the explanations provided by the reasoner (highlighted green text) for those inferences (valid tumour signature, invalid grade, and valid basis of diagnosis).

Figure 2 shows the validation results for a hypothetical cancer case with the given class assertions. The reasoner has inferred that: (a) the tumour signature is a valid combination of morphology and topography codes; (b) the grade is incorrectly coded; and (c) the basis of diagnosis is valid. The first reasoner trace immediately below the class description window shows that the tumour signature instance c051_m8082 is defined by an existential relation with the morphology class M_8082 and an existential relation with the topography class C051. The reasoner uses the fact that the M_8082 class is subclassed from the C051Morph surrogate topography class (c.f. Figure A1 of the Appendix A) to infer that the existential relation with M_8082 is subsumed by an existential relation with C051Morph. It then uses the axiom highlighted with the blue background to subsume the tumour signature instance c051_m8082 under the VALID_TumourSignature class, c.f. Equation (7).

The second reasoner trace in Figure 2 provides the explanation for an invalid grade inference. From the predefined equivalence statement in the ontology, the reasoner recognises that the asserted value of grade is associated with a haematological morphology. The asserted morphology code is however subclassed from a non-haematological morphology. The axiom highlighted with the blue background is a rule that states a haematological-type grade with a non-haematological morphology is subsumed under an invalid grade condition. 

The final reasoner trace relates to a valid basis for diagnosis inference. In this case, the inference is trivially determined since it is the default case for the basis of diagnosis involving a histological examination of an excised sample of the tumour (basis of diagnosis code 7). The reasoner infers the valid basis of diagnosis default case from the asserted basis of diagnosis code (7) and the associated default rule highlighted with a blue background in the window.

### 3.2. Validation Results Using the TNM-Specific Ontology (TNM_CR_O)

**Figure 3:**
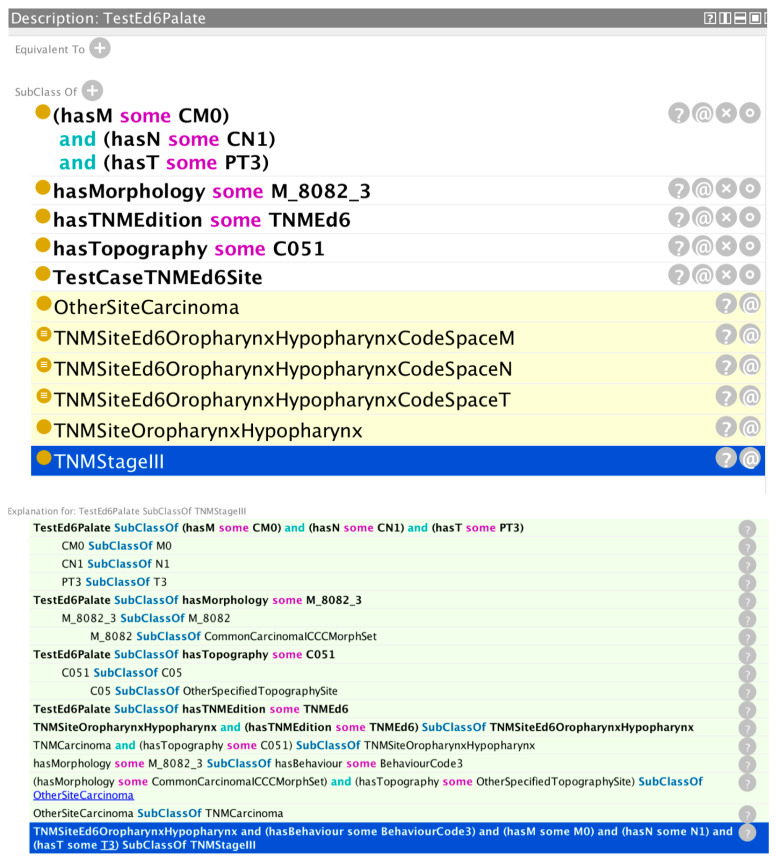
Validation results for a hypothetical cancer case with TNM data. The reasoner has correctly inferred stage group III (top window, bottom line highlighted with a blue background).

Figure 3 shows the validation results for a hypothetical cancer registry case with TNM data.

The reasoner has correctly inferred a stage group III based on the values asserted in the tumour case (morphology, topography, TNM edition, and T, N, and M parameters). The trace shows how the reasoner has determined a morphology group of common carcinomas from the asserted morphology and behaviour codes. Likewise, it has determined a topography site of “other specified site” from the asserted topography code. It then uses this information to find a subsumption under the class OtherSiteCarcinoma, which is itself a subclass of the TNMCarcinoma class, and this, together with the asserted topography class, results in a class subsumption under TNMSiteOropharynxHypopharynx—which for the asserted TNM edition results in a class subsumption under the TNMEd6 associated class. Finally, with the additional information of the asserted T, N, and M values, the reasoner infers a stage value of III from the rule highlighted with a blue background on the last line of the reasoner trace window.

### 3.3. Validation Results Using the Multiple Primary Tumour Ontology (ENCR_MPT_O)

**Figure 4:**
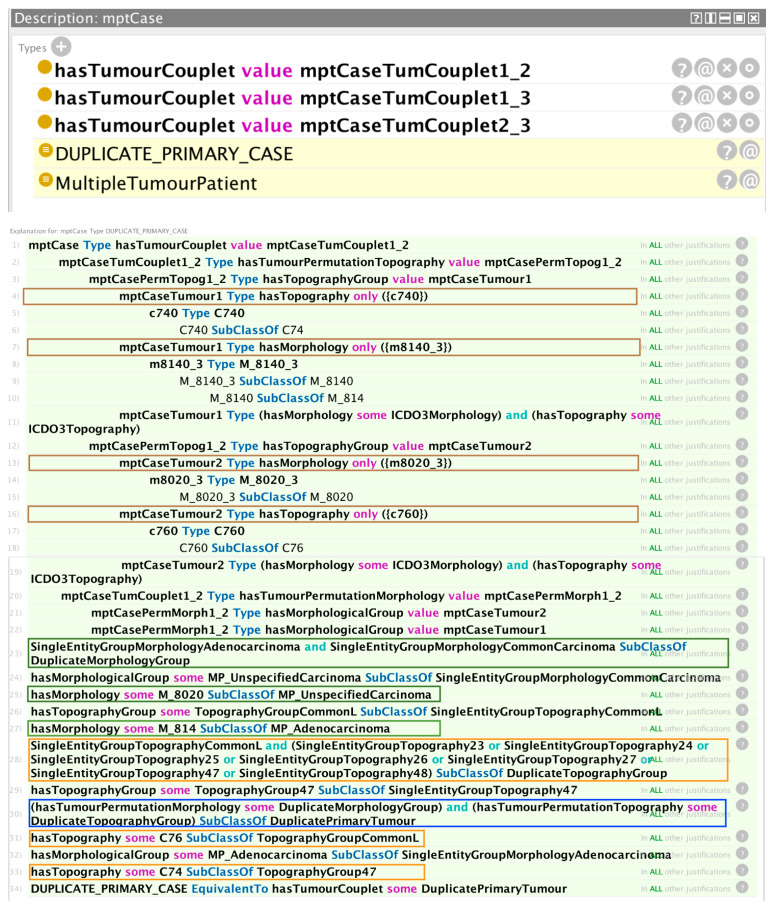
Example of a hypothetical multiple primary cancer case in which the reasoner has determined the occurrence of a duplicate primary tumour in one of the three tumour couplets.

Figure 4 is an example of a hypothetical multiple primary cancer case with three associated tumours, for which the reasoner has detected a violation of a condition for independent primary tumours. The asserted axioms are constructed using the three unique pairwise permutations (tumour couplets) of the three diagnosed tumours (tumours 1 and 2; tumours 1 and 3; and tumours 2 and 3, each corresponding to the three tumour couplet assertions shown in the figure).

The reasoner trace window provides the explanation for the rule violation inference with reference to the defaulting pair of tumours (the tumour couplet involving tumours 1 and 2). The first four boxed statements show the asserted topography and morphology values of the two tumours. The next three boxed statements show that the morphologies of the two tumours belong to a common morphology group. One of the morphologies belongs to the set of adenocarcinomas and the other belongs to the set of unspecified carcinomas; however, in combination, these two sets fall under a common morphology group, as shown. The three orange-boxed statements show that the topographies of the two tumours also belong to a common group. Tumours having morphologies and topographies both in common groups satisfy one of the conditions of a duplicate primary condition, as encapsulated in the axiom boxed by the blue rectangle. The equivalence statement on the last line leads the reasoner to infer a duplicate primary case, as indicated in the top window of Figure 4.

### 3.4. Metrics and Performance of the Ontology Modules

The main sets of metrics describing the ontology modules may be found in Table A2 of the Appendix A. The table includes the three main ontologies as well as a variety of composite ontologies incorporating other specific modules or main ontologies according to the modular design we used to allow such integration. The table also provides a comparison of reasoning performance between the four different reasoners we investigated (FaCT++, HemiT, Pellet, and ELK), all of which are freely distributed with Protégé.

## 4. Discussion

Ontologies are a powerful knowledge-organisation tool, and while data validation is not their primary function, they are able to support it via their foundation in description logics. The process of designing an ontology, however, is not an easy one, and ontology engineering is an emerging discipline; the ontology engineer must not only have an adequate understanding of the domain itself but be aware of the sorts of constraints that may influence the design of the axioms. Ontology development is seen as an iterative process [14] requiring frequent invocation of the reasoner; seemingly innocent changes can have major impacts on the speed of reasoning [35], and reasoning performance is also dependent on the reasoner used. 

### 4.1. Main Findings

We have been able to show that ontology-based AI applications can successfully handle the majority of the ENCR validation rules for cancer registry data, even for quite complex inter-variable conditions. Using certain design patterns, we were able to overcome some of the DL restrictions that generally make DL reasoning challenging in applications more suited to the closed-world assumption. Other design patterns allowed us to limit the expressivity and reduce performance overheads. Regarding the reasoners we investigated, we found that FaCT++ and Pellet were more sensitive than HermiT to small changes in the axioms that disproportionately affected performance. Pellet performed consistently worst of all for reasons that appeared to be more associated with CPU load than memory space. While ELK performed consistently well, it is limited to a restricted set of operations and could not be used for validating the multiple primary tumour data. FaCT++ resulted in the best performance in the latter case.

### 4.2. Implications

Reasoning performance is an important factor to consider for any ontology-based AI application. Performance issues are, however, generally only noticeable for ontologies with more than several thousand classes, by which time it is more difficult to change the ontology structure. There are few guidelines available to help steer design choices, and the choice may, in any case, be constrained by the application domain. The requirements of data validation do impose certain restrictions on the design of the ontology, and we have highlighted a few issues and proposed some design patterns to help tackle them. One particular example is in relation to the open-world assumption of DLs; in order to force the necessary class subsumption requirements, we had to make extensive use of GCI constructs. GCIs can be expensive operations [36], but it is not otherwise possible to achieve the necessary containment. Moreover, the GCI construct is not so well supported in the Protégé editor (either in terms of text searching or visualisation of the asserted rules) and the value of the tool could be considerably enhanced were this functionality to be further developed. Reasoning performance is also an issue for ontologies with large ABoxes, and cancer registry data sets can run up to hundreds of thousands and even millions of records.

Designing an ontology using an appropriate modular architecture allows some performance gains on the basis of reasoner performance. For example, FaCT++ performed better for the multiple primary tumour ontology (ENCR_MPT_O) than the other reasoners we used, while HermiT was faster for the TNM ontology (TNM_CR_O), c.f. Table A2. Limiting expressivity to εL allows reasoning to be performed in polynomial time [35] (ELK is able to classify the 300,000 classes of SNOMED in the space of a few seconds [37]). The limitations of εL expressivity are not unduly restrictive for many types of validation checks, and ontologies can be designed carefully to respect the constraints. Only two particular types of checks in the ENCR validation process (age checks and multiple primary tumour checks) require greater expressivity and could be handled separately using other reasoners. Optimisation of reasoning algorithms remains an active field of research. 

Other performance gains could be achieved via parallelisation. Validation is generally performed on a record-by-record basis, and the task is therefore highly parallelisable, serving to overcome performance bottlenecks on a single processor. The validation tests could themselves also be serialised and passed to the relevant ontology suite, c.f. Table A2, thus providing further motivation for a modular-based ontology design.

### 4.3. Motivations for the Ontology Approach to Data Validation

A major advantage of using ontologies for data validation is that of being able to maintain both the validation rules and the intelligence of the validation checks within the ontology itself. One need only invoke the reasoner on the basis of those axioms to validate any particular data. While this can be performed on a case-by-case basis using an ontology editor such as Protégé, complete data sets can be tested via the OWL application programming interface (OWL-API) [38]. The interface programme needs only to read in the individual records of a data set, translate them to the DL statements required by the ontology, and then invoke the reasoner. The application programme thus becomes more of a data-housekeeping task. The OWL-API can nevertheless also be used to perform the logic operations in the software and transfer the intelligence from the ontology to the application programme where necessary. Although this does have an impact on development and maintenance costs, it provides a means for overcoming limitations due to reasoning performance whilst still benefiting from the logical axioms defined in the ontology.

The unification of these aspects within a single application is attractive for the eventual devolution of centralised data-cleaning processes to the local cancer registry level. Application of a consistent and standard approach has been one of the hurdles in the past since it is critical that no biases are introduced from an inconsistent application of the validation rules. A further advantage of the ontological approach is that it allows some quantifiable and reproducible means of measuring several quality metrics of data sets, affording users greater assurance in the comparability/integration of data sets from different sources [39].

Ontologies are a useful tool allowing quick comparison and reference across multiple classification schemes, which can also serve to highlight where code hierarchies could be arranged more efficiently. For example, we had to define many different classification trees for the morphology codes (dependent on the specific validation checks). This resulted in considerable entanglement in the asserted classification trees due to multiple inheritance and conveys the need for greater rationalisation in morphology classification. 

### 4.4. Limitations

In addition to reasoning performance issues, it must also be admitted that ontologies are not necessarily able to handle all types of validation checks; some notable exceptions are those that require the comparison of dates and the tracking of frequencies of variables. Such checks within the ENCR data, however, can be handled without undue difficulty in a pre-processing stage. The latter is, in any case, necessary for transcribing the raw data files into RDF prior to insertion into the ontology. Alternatively, there are other useful tools within the semantic web stack that can perform checks less suited to ontologies. The shape language ShEx is able to trap range and format errors and SPARQL scripts provides a versatile means for determining, amongst other things, the frequencies of variables in a given data set. The interesting aspect from the perspectives of maintenance and process harmonisation is that the tools all work on RDF data, which is one of the default representational formats of OWL ontologies.

### 4.5. Other AI-Based Approaches and Future Directions

Deep learning techniques could undoubtedly be used to validate data, and a commercial product along these lines is available for validating the accuracy of clinical trial results [40]. Such an approach has advantages in terms of reasoning speeds but requires a specific set of skills for developing and maintaining the associated application. Our reason for considering an ontological approach was to provide a range of functionalities, including: (a) a knowledge base allowing the integration of all the semantic relationships defined over a number of different classification schemes (ICD-O, TNM, and ICCC); (b) a means of formalising the data rules; (c) a means of defining and linking the metadata; and (d) a single application suite with a standard interface.

There is, however, no reason why the two approaches could not be used together, and a powerful symbiosis exists between extract, transform, and load (ETL) approaches and semantic representations of ontologies. The semantic relations, and even the rule base itself, could be derived and transformed using ETL into DL axioms from the whole of the European cancer registry data and stored in an ontology. The ontology could, thereafter, be used by any machine-learning process for data validation purposes.

More immediate aims to help consolidate ontology-based AI in the data validation domain would be to identify and optimise a full range of generic design patterns. It would be useful also to understand the underlying reasons for the formation of reasoning performance hotspots [35] and to find approaches/design patterns for avoiding them, as well as to deploy advanced reasoning tools as and when they become available. 

## 5. Conclusions

Ontology-based AI can provide a number of advantages over traditional approaches to validating cancer registry data. Ontologies are able to describe the necessary conceptual and relational information associated with a given domain and express the dependencies in formal logic. The subsequent integration of the validation checks within the knowledge base means that any modifications to the rules base can be kept in synchronisation with the data model itself. This, in turn, simplifies maintenance issues and facilitates the federation of the validation process, thereby overcoming many of the hurdles associated with centralised data collection. Data validation is, however, not the primary purpose of ontologies, and the associated idiosyncrasies impose certain design constraints and patterns on the ontology structure for AI-based reasoning that are not immediately obvious. The design patterns and constraints presented here are generic to other domains beyond cancer registries and serve to point out the types of issues validation ontologies have to confront, with proposed solutions for tackling them. Discovery and optimisation (especially in overcoming reasoning hotspot issues) of other such patterns would provide an important and valuable contribution to the advancement of ontology-based AI for data validation purposes. It is hoped that this study may give impetus to further work in this area, which is relevant for all data domains.

## Data Availability

The ontologies are available as follows: ENCR core-validation ontology: http://data.europa.eu/89h/efd6acd1-2cfd-401b-b5ea-d05f8efbb123 (accessed on 8 December 2023); stage-validation ontology: http://data.europa.eu/89h/9fa603ff-a118-41f3-82a2-bf8d4f0d7ea3 (accessed on 8 December 2023); ICD-O-3 ontology: http://data.europa.eu/89h/88ff4ec5-1832-403e-abe1-64928592568f (accessed on 8 December 2023); ICCC-3 ontology: http://data.europa.eu/89h/6f69e886-e5bc-4cd8-9b2d-8aaccf836789 (accessed on 8 December 2023); and multiple primary tumour-validation ontology: http://data.europa.eu/89h/2a110a2e-d1e2-439d-9b9f-7e2e5436cc49 (accessed on 8 December 2023).

## Abbreviations

AI	artificial intelligence
DL	description logic
ECIS	European cancer information system
ENCR	European Network of Cancer Registries
ENCR_QC_O	ENCR quality checks ontology
ETL	extract, transform, and load
GCI	general concept inclusion
ICCC	International classification of childhood cancer
ICCC3_O	ICCC3 ontology
ICD-O	International classification of diseases for oncology
ICDO3_O	ICD-O-3 ontology
OWL	web ontology language
OWL-API	OWL application programming interface
TNM	classification standard of malignant tumours
TNM_CR_O	TNM cancer registry ontology

## Appendix A

**Table A1:** Summary of mechanisms described in Section 2.3 that can be employed to ensure the desired class subsumption results and that overcome some of the inherent constraints of DLs in closed-world reasoning.

DL Constraint	Mechanism to Circumvent Constraint Whilst Limiting Expressivity	Example	Axiom(s)
open-world assumption	GCI (to force the required class subsumption by subclassing the axiom under the desired outcome class)	behaviour	∃hasMorphology.M_8082_3 ⊑ ∃hasBehaviour.BehaviorCode3 (the existential relation ∃hasMorphology.M_8082_3 is automatically subsumed under the existential relation ∃hasBehaviour.BehaviorCode3, allowing other axioms to deduce that the existential relation with the class M_8082_3 is associated with the malignant behaviour code 3)
open-world assumption and inverse roles	GCI and “surrogate” classes	tumour signature	M_8082 ⊑ C051Morph (subclassing of morphology class under the associated “surrogate” topography class) ∃hasMorphology.C051Morph ⊓ ∃hasTopography.C051 ⊑ VALID_TumourSignature (subsumption by a valid condition class of the intersection of the real topography class and the surrogate topography class, which links in all the subclassed morphology codes)
role chaining	subclassing a class with a name qualifier under a class of the same name without the qualifier, and using a GCI to force the qualified relation	behaviour	M_8082_3 ⊑ M_8082 (subclassing of behaviour-flagged morphology class under the corresponding morphology class) ∃hasMorphology.M_8082_3 ⊑ ∃hasBehaviour.BehaviorCode3 (c.f. axiom in the first row of the table. The axioms avoid having to define a chain role such as hasMorphology ∘ hasBehaviour ⊑ hasBehaviour)
monotonicity	default condition and secondary override condition	basis of diagnosis	∃prevalidatedBoD.BoDcode2 ⊑ InvalidBoDDefaultCase (subclassing a prevalidated class under a default class) ∃prevalidatedBoD.BoDcode2 ⊓ ∃hasMorphology.M_8000_1 ⊓ ∃hasTopography.C751 ⊑ VALID_BoD (subsuming the intersection, under a class with the opposing validity to the default value, of the prevalidated class with the set of conditions that serve to invalidate the default condition)

**Table A2:** Summary of the metrics for the ontologies and their various integrations into larger containing ontologies. The attribute ^(D)^ in the language expressivity refers to the use of datatype properties. An asterisk against the reasoning time in the last column indicates that any datatype expressions in the associated ontologies are ignored by the ELK reasoner.

Ontology	Expressivity	Logical Axioms	Class Count	Object Properties	Data Properties	SubclassOf	Defined Class	GCI Count	Reasoning Times (Seconds)			
									FaCT++	HermiT	Pellet	ELK
ICDO3_O	ALC	3971	2753	9	0	3948	11	1202	<1	<1	<1	<1
ICCC_O	ALC	6651	2891	5	0	6646	1	3465	<1	<1	7	<1
Tumour Signature (TS)	ALC	7661	3157	3	0	7654	1	1533	2	8	11	<1
ENCR_QC_O without TS	ALC^(D)^	8428	2964	12	4	8349	11	5013	2	2	57	1 *
ENCR_QC_O	ALC^(D)^	12,155	3392	12	4	12,074	11	5344	3	14	175	2 *
ENCR_MPT_ O	ALCOIQ^(D)^	7966	3129	9	1	7399	15	3891	2	23	>900	N/A
TNM Ed6	ALCI^(D)^	8926	3410	21	1	8682	141	4688	4	2	10	1 *
TNM Ed7	ALCI^(D)^	9409	3501	21	1	9100	204	4939	5	2	20	1 *
TNM_CR_O	ALCI^(D)^	10,740	3684	21	1	10,302	332	5777	15	3	53	1 *
CCS_TGL_O	ALC^(D)^	7536	3358	23	0	7409	22	3544	2	2	12	<1 *
ENCR_QC_O without TS + TNMEd7	ALCI^(D)^	10,956	3619	24	4	10,588	204	6269	6	4	90	1 *
ENCR_QC_O + TNMEd7	ALCI^(D)^	14,683	4002	24	4	14,313	204	6600	9	10	450	1 *
ENCR_QC_O without TS + TNM_CR_O	ALCI^(D)^	12,287	3757	24	4	11,790	332	7107	17	5	240	1 *
ENCR_QC_O + TNM_CR_O	ALCI^(D)^	16,014	4185	24	4	15,515	332	7438	19	14	540	2 *
ENCR_QC_O + CCS_TGL_O	ALC^(D)^	13,386	3834	24	4	13,201	22	5807	3	11	165	1 *
